# Difficult tracheal intubation in a patient with maternal uniparental disomy 14

**DOI:** 10.1186/s40981-016-0051-8

**Published:** 2016-09-26

**Authors:** Kenta Furutani, Yoshie Kodera, Masataka Hiruma, Hideaki Ishii, Hiroshi Baba

**Affiliations:** Division of Anesthesiology, Niigata University Medical and Dental Sciences, 1-757 Asahimachi-Dori, Chuo-ku, Niigata 951-8510 Japan

**Keywords:** Maternal uniparental disomy 14, Difficult intubation, General anesthesia

## Abstract

**Background:**

Maternal uniparental disomy 14 (UPD(14)mat) is an imprinting disorder. It is a rare disease, but there is the possibility that more undiagnosed patients might exist because the clinical features of UPD(14)mat resemble those of the Prader-Willi syndrome or other congenital diseases. We performed anesthetic management for an 8-year-old girl with UPD(14)mat.

**Case presentations:**

She was admitted to undergo correction surgery due to symptomatic scoliosis. Preoperative examination revealed that she had a restricted mouth opening and retrognathia, as well as some typical characteristics of UPD(14)mat, such as small hands, growth retardation, and precocious puberty. We induced general anesthesia using sevoflurane without any problems. However, the tracheal intubation was difficult because of the restricted mouth opening. We used the McGRATH^R^ MAC videolaryngoscope to overcome this problem.

**Conclusions:**

We speculate that the craniofacial deformity in case of UPD(14)mat patients may lead to difficulty in tracheal intubation.

## Background

Uniparental disomy (UPD) is the condition in which an individual carries two homologues of a chromosome pair originating from one parent and none from the other. Maternal UPD 14, expressed as “UPD(14)mat,” means that both chromosomes 14 originate from the mother.

Maternal uniparental disomy 14 (UPD(14)mat) is known as one of the genomic imprinting disorders. An imprinted gene is one which acts at the time of either the father origin (paternally expressed gene) or the mother origin (maternally expressed gene). Therefore, UPD results in either overexpression or deletion of the imprinting genes. As a result, a patient with UPD(14)mat has the characteristic symptoms that are different from those of a patient with paternal UPD 14. Clinical features of UPD(14)mat include pre- and postnatal growth retardation, neonatal hypotonia, small hands and feet, feeding difficulty, and precocious puberty [1].

There are no reports about anesthetic management for a patient with UPD(14)mat. Therefore, we report the case of a patient with UPD(14)mat, in which we faced difficulty in tracheal intubation because of restricted mouth opening and retrognathia.

## Case presentation

We obtained a written consent for publication of this case from the patient’s parent.

An 8-year-old girl was diagnosed as symptomatic scoliosis. She was admitted to our hospital to undergo growing rod application under general anesthesia.

The patient was born as a low birth weight baby (1800 g). Because she had Prader-Willi syndrome (PWS)-like symptoms, such as feeding difficulty and hypotonia, a chromosome banding analysis had been carried out and she had been diagnosed as having UPD(14)mat. Because of growth retardation, growth hormone (GH) replacement therapy was started when she was 3 years old but was discontinued because her scoliosis was pointed out when she was 4 years old. She was also diagnosed as having precocious puberty when she was 6 years old, and luteinizing hormone-releasing hormone (LH-RH) analogue therapy was started.

A preoperative physical examination revealed her height to be 113.4 cm (−1.7 SD) and her weight 16.2 kg (−2.0 SD). She had small hands and feet. Her intelligence was almost normal. Her Cobb angle was 79°. We evaluated that she had two problems with regard to airway management: first, she had a restricted mouth opening (11 mm) and retrognathia; and second, the pediatrician suggested the possibility of adenoidal or tonsillar hypertrophy due to the influence of GH replacement therapy.

We prepared LMA ProSeal (Teleflex, Inc., Wayne, PA, USA) and McGRATH MAC videolaryngoscope with a size 2 blade (Aircraft medical, Edinburgh, UK) for the suspected difficult airway. General anesthesia was induced by inhalation of 5 % sevoflurane with a mixture of 40 % oxygen and 60 % nitrous oxide. Mask ventilation was easy. After an intravenous line was secured and rocuronium 10 mg was administered, we tried tracheal intubation using Macintosh laryngoscope. However, we failed twice due to restricted mouth opening and retrognathia. Glottic view during laryngoscopy was classified as Cormack-Lahane Grade 3. Finally, McGRATH MAC videolaryngoscope with a size 2 blade enabled us to view the glottis and to intubate the trachea successfully. Thereafter, sevoflurane was stopped, and a continuous infusion of propofol and remifentanil was started to record motor- and somatosensory-evoked potentials. We faced no problems in the intraoperative management. After emergence from general anesthesia, extubation was performed smoothly. The operation time and the anesthesia time were 196 and 382 min, respectively. Postoperative analgesia was administered by intravenous patient-controlled analgesia with continuous infusion of fentanyl. As a result, she experienced little pain postoperatively. After the surgery, her scoliosis improved, her Cobb angle was 33°, and she was discharged on the tenth postoperative day.

The present case highlighted two clinical issues. This is the first case report of the anesthetic management of a patient with UPD(14)mat, and we faced difficulty in performing laryngoscopy due to the restricted mouth opening with retrognathia.

UPD(14)mat is known as a rare disease. However, there is the possibility that more undiagnosed patients might exist. It was reported that 5 of 78 patients who presented with PWS-like phenotype without PWS-specific genetic abnormality had UPD(14)mat or 14q32.2 epimutation [2]. In addition, the features of UPD(14)mat resemble PWS or the other congenital diseases [1]. Therefore, it is important that anesthesiologists recognize the problems that can occur during the anesthetic management of patients with UPD(14)mat.

UPD(14)mat is known as one of the genomic imprinting disorders. It was reported that the imprinted locus of UPD(14)mat was in 14q32.2 [3]. Imprinting disorders include PWS, Silver-Russell syndrome, Beckwith-Wiedemann syndrome, Angelman syndrome, and neonatal transient diabetes mellitus [4]. Because medical treatment for UPD(14)mat has not been established, symptomatic treatments, for example, GH replacement therapy for short stature, and LH-RH analogue therapy for precocious puberty, are important.

Possible concern about the anesthetic management for a UPD(14)mat patient is airway management. We faced difficulty in performing laryngoscopy due to the restricted mouth opening with retrognathia in our patient. It has been reported that less than 40 % of patients with UPD(14)mat have scoliosis [5], and general anesthesia is necessary for the correction surgery. Although the association between UPD(14)mat and restricted mouth opening is unclear, craniofacial deformity (high arched palate, frontal bossing, short philtrum, mild blepharophimosis, high forehead) is found in patients with UPD(14)mat [6]. We speculate that this craniofacial deformity might lead to difficulty in tracheal intubation. Although we could intubate her trachea with McGRATH MAC, fibreoptic tracheal intubation might be considered for a UPD(14)mat patient with restricted mouth opening. In addition, precocious puberty is a characteristic feature of UPD(14)mat and contributes to short stature without the treatment such as LH-RH analogue therapy. GH replacement therapy for the treatment of short stature might cause adenoidal or tonsillar hypertrophy. Therefore, a UPD(14)mat patient might have difficulty in both tracheal intubation and mask ventilation. The anesthesiologists should prepare for difficult airway when they would manage a UPD(14)mat patient.

We also should pay attention to the other features of UPD(14)mat, for example, joint hypermobility, psychomotor delay, or truncal obesity [5]. More than one third of patients has mild to moderate mental retardation, although intelligence is within normal range in the majority of them [5].

## Conclusions

We described the anesthetic management for scoliosis surgery of a patient with UPD(14)mat, in which tracheal intubation was a problem because of the restricted mouth opening with retrognathia. Further reports should be accumulated to determine whether those clinical features of UPD(14)mat may be related to difficult tracheal intubation.
